# Down-regulation of S100A9 inhibits osteosarcoma cell growth through inactivating MAPK and NF-κB signaling pathways

**DOI:** 10.1186/s12885-016-2294-1

**Published:** 2016-03-28

**Authors:** Si Cheng, Xi Zhang, Ning Huang, Quanhe Qiu, Ying Jin, Dianming Jiang

**Affiliations:** Department of Orthopaedics, The First Affiliated Hospital of Chongqing Medical University, Chongqing, PR China; Department of Nephrology, The First Affiliated Hospital of Chongqing Medical University, Chongqing, PR China; Department of Neurosurgery, The Second Affiliated Hospital of Chongqing Medical University, Chongqing, PR China; Institute of Life Sciences,Chongqing Medical University, Chongqing, PR China; Department of Orthodontics, State Key Laboratory of Oral Diseases, West China Hospital of Stomatology, WestChina School of Stomatology, Sichuan University, Chongqing, PR China

**Keywords:** S100A9, Osteosarcoma, Proliferation, Invasion, Tumorigenesis, MAPK, NF-κB

## Abstract

**Background:**

Osteosarcoma (OS) is well-known for poor prognosis due to its high incidence of proliferation and metastasis. Researches have provided valuable insights into the tumorigenesis of S100A9 in some cancers. We aimed to understand the expression level, functions and mechanisms of S100A9 in human osteosarcoma for the first time.

**Methods:**

The expression of S100A9 protein was detected in 120 human osteosarcoma tissues and 40 normal human bone tissues using tissue microarrays analysis. The knockdown of S100A9 induced by RNA interference (RNAi) method in three osteosarcoma cell lines (U2OS, 143B, MG63) was applied to analyze the effects of S100A9 on cell proliferation, cell cycle distribution, migration, invasion and xenotransplanted tumors. Moreover, MAPK-ERK1/2, MAPK-p38, NF-κB-p65, NF-κB-p50, p21, p27, CDK2 and CDK4 were tested.

**Results:**

The expression of S100A9 was increased in human osteosarcoma issues and was positively correlated with clinical classification and survival rate. Down-regulation of S100A9 inhibited OS cellular proliferation, migration, invasion and cell cycle S phase in vitro and suppressed tumor formation in vivo with the reduction on PCNA and Ki67 proliferation index. Our data also demonstrated that knockdown of S100A9 repressed the protein levels of phospho-ERK1/2, phospho-p50, phospho-p65 except phospho-p38, and prompted up-regulation of p21 and p27 leading to inactivation of cyclin dependent kinase 2(CDK2) and cyclin dependent kinase 4(CDK4).

**Conclusions:**

S100A9 might be a significant role for predicting osteosarcoma prognosis and down-regulation of S100A9 could be used as a potential target for gene therapy.

**Electronic supplementary material:**

The online version of this article (doi:10.1186/s12885-016-2294-1) contains supplementary material, which is available to authorized users.

## Background

Osteosarcoma (OS) is one of the predominant bone sarcomas [1] as well as the third most common primary malignant bone tumor happened in children and adolescents [2]. OS is commonly characterized by its aggressive growth, high rate of local recurrence, and poor long-term survival rates for the early frequent systemic metastases, particularly for the lung metastasis [3]. Currently, the 5-year survival rate for patients with local OS remains approximately 65–70 % and for patients with metastatic diseases merely remains 20 %. Over the past 15 years, only a slight improvement was made in terms of OS therapeutic effects [4]. The main clinical treatment for OS patients includes wide surgical removal of all detectable disease (including metastases) and pre- or post-operative chemotherapy [5]. However, current chemotherapies often result in systemic toxicities (hearing loss, anemia, abnormal bleeding, and kidney/liver damage) and chemoresistance [6, 7]. Thus, the OS treatment requires the development of some new-targeted therapies. Predicting the possibilities of proliferation or metastases in the OS patients would offer more options for doctors to make a suitable therapeutic strategy. A few new biomarkers have been found, and these biomarkers can be considered as the targets for ensuring effectiveness of the OS treatment, contributing to a better clinical management for OS patients.

S100A9 (calgranulin B or MRP-14), known as damage-associated molecular pattern (DAMP) molecule, is secreted by myeloid cells upon activation [8]. The intracellular and extracellular functions of S100A9 include calcium sensing, activation of NADPH oxidase and arachidonic acid transport [9, 10], regulation of tubulin-dependent cytoskeletal rearrangements [11] and effecting on cell migration and adhesion [12]. S100A9 was found in inflammatory conditions as well [13, 14]. The relation between inflammation and carcinogenesis has long been recognized [15]. Increasing evidences affirmed that S100A9 plays an important role in tumorigenesis. The up-regulation of S100A9 has been observed in colon, gastric, bladder, pancreatic, ovarian, breast thyroid, and skin cancers [16], while S100A9 is reduced in in esophageal squamous cell carcinoma [17].

Mitogen-activated protein kinase (MAPK) is an insulin-mitogen activated protein (Ser/Thr) kinase [18]. It is associated with cellular growth, survival and migration through regulating the signals from cell-surface to the nucleus by phosphorylation [19]. NF-κB, a transcription factor-a cytoplasmic heterodimer or homodimer interacting with an inhibitory protein of the IkB family, plays a critical role in the promotion of tumorigenesis [20, 21]. Some articles have reported that the activation of MAPK and NF-κB signaling pathways were detected in tumor cells and these two signaling pathways could be significance directions for oncotherapy [22, 23].

In this study, we investigated the expression of S100A9 protein in human osteosarcoma clinical samples and analyzed relevant clinicopathological characteristics. The functions of S100A9 were further understood in OS cells. Our results demonstrated that the knockdown of S100A9 inhibited human OS cellular growth in vitro and in vivo. To the best of our knowledge, this is the first report stating that over-expression of S100A9 might be a prerequisite for development and progression in human osteosarcoma.

## Methods

### Reagents

Fetal bovine serum (FBS) and Dulbecco’s modified Eagle’s medium (DMEM) were purchased from Gibco (San Francisco, California, USA). Primary anti-bodies: rabbit anti-human S100A9 was bought from ABCAM (MA, USA). Rabbit anti-human total ERK1/2 MAPK, rabbit anti-human total p38 MAPK, rabbit anti-human total p50 NF-κB, rabbit anti-human total p65 NF-κB and antibodies against phospho-ERK1/2 MAPK, phospho-p38 MAPK, phospho-p50 NF-κB, phospho-65 NF-κB were bought from Cell Signaling Technology (Boston, Massachusetts, USA). Rabbit anti-human p21 and p27 were purchased from Anbo Biotechnology (San Francisco, California, USA). Rabbit anti-human Ki67 nuclear antigen, mouse anti-human PCNA and mouse anti-human glyceraldehydes-3-phosphate dehydrogenase (GAPDH) were purchased from Santa Cruz Biotechnology (San Francisco, California, USA). Horseradish peroxidase-conjugated goat anti-rabbit and goat anti-mouse secondary antibodies were purchased from Zhong Shan Golden Bridge Biotechnology (Beijing, China).

### Tumor samples

A total of 120 osteosarcoma (OS) patients who came from the First Affiliated Hospitals of Chongqing Medical University, Second Affiliated Hospitals of Chongqing Medical University, Children’s Hospital of Chongqing Medical University (Chongqing, China) and Tumour hospital of Guizhou (Guizhou, China) between 2005 and 2014 were enrolled in this study. All tumor biopsies were collected at the time of initial diagnosis prior to preoperative chemotherapy or radiotherapy, with informed consent from patients/guardians. The patients were divided into IA, IB, IIA, IIB and III grades according to the GTM staging system (G-Histologic Grade, T-Anatomic site, M-Metastasis). This study was approved by the ethics committee of the First Affiliated Hospitals of Chongqing Medical University, Second Affiliated Hospitals of Chongqing Medical University, Children’s Hospital of Chongqing Medical University (Chongqing, China) and Tumour hospital of Guizhou (Guizhou, China).

### Normal bone samples

A total of 40 normal bone tissues that came from the First Affiliated Hospitals of Chongqing Medical University were enrolled with consent from patients/guardians between 2012 and 2014. This study was approved by the ethics committee of the First Affiliated Hospitals of Chongqing Medical University.

### Tissue microarrays

Tissue microarrays (TMAs) were constructed from 120 paraffin blocks of tumor tissues and 40 normal bone tissues using a tissue array device (Beecher Instruments, Sun Prairie, WI).

### Immunohistochemistry (IHC)

Antigen retrieval on the deparaffinized sections was performed by immersing the samples in 0.1 M citrate buffer (pH 6.0), boiling the sections in the microwave for 10 min, and then allowing the sections to cool down to room temperature. Endogenous peroxidase activity was blocked by immersing the sections in methanol containing 3 % hydrogen peroxide for 10 min. After blocked in goat serum for 10 min at room temperature, the sections were incubated with the S100A9 antibody (1:50) overnight at 4 °C. Then, the sections were incubated with the secondary antibody at 37 °C for 30 min. Streptavidin conjugated peroxidase was added and kept for 10 min at room temperature. Diamino-benzidine substrate was added and kept for 5 min for visualizing. Immunohistochemical staining of S100A9 provided calculations of both percentage of positive cells and color intensity. The percentage of the positivity of staining was graded as “0”(negative), “1”(<10 %), “2” (10–50 %), and “3”(>50 %). The intensity of staining was scored as “0” (absent), “1”(light yellow),“2”(yellowish brown) and “3”(brown). The staining index(SI) was used for assessing the expression of S100A9 protein. According to SI = proportion score × intensity score, 0 was categorized as negative(-); 1–2 as low expression(1+); 3–4 as moderate expression(2+); 6 and 9 as high expression(3+).

### Cell culture

Human OS lines MG63,143B and U2OS were recently purchased from Shanghai Life Academy of Sciences cell library (Shanghai, China). The OS lines (MG63,143B,2OS) were maintained in DMEM containing 10 % fetal bovine serum, 100U/ml penicillin and 100U/ml streptomycin (Hyclone, Longan, Utah, USA) at 37 °C in 95 % air/5 % CO_2_ incubator_._

### Knockdown of S100A9 in OS cells

The siRNA sequences targeting S100A9 (AGGAGTTCATCATGCTGAT) were purchased from Invitrogen. OS cells in the exponential phase of growth were plated in six-well plates at 1 × 10^5^ cells/well, incubated for 24 h, then OS cells were transfected with 2 μg of each plasmid for 6 h using Lipofectamine 2000 reagent (Invitrogen) and OPTI-MEM reduced serum medium (Invitrogen). The control OS cells were transfected with empty vectors. After 48 h, the OS cells were incubated in a complete medium containing puromycin dihydrochloride (Sigma-Aldrich Co. St Louis, MO, USA; 2 μg/ml for 143B, MG63 and U2OS cell lines) for 2 weeks and subcloned the individual Puromycin-resistant cells in 6 well plates and expanded the cells into puromycin-resistant sublines.

### Measurement of cellular proliferation

OS cells were seeded in 96-well plates at a density of 2000 cells/well. 10ul CCK-8(Beyotime Institute of Biotechnology, Beijing, China) was added to each well after 24 h, 48 h, 72 h and 96 h. The absorbance value was readable at 450 nm using an enzyme- labeled instrument.

### Flow cytometry analysis (FCM) of cell cycle distributions

5 × 10^5^ cells were harvested and fixed in 70 % ice-cold ethanol at 4 °C overnight. The cells were incubated with 10 mg/ml RNase (Sigma, St Louis, Missouri, USA) and 50 μg/ml propidium iodide(Sigma) at 37 °C for 30 min in the dark. The cell cycle was analyzed by flow cytometer (BD Bioscience, Franklin Lakes, New Jersey, USA).

### Cell migration assay

The migration of OS cells was assayed using the transwell chambers (BD Biosciences, CA, USA). The back of chambers was precoated with 5 mg/ml fibronectin (50 μl). The layer of fibronectin should be dried for 1 h. The OS cells (1 × 10^5^) were put in the upper side of the 8-μm pore size transwell chambers containing 0.1 ml DMEM without serum and 500 μl DMEM containing 10 % FBS were added in 24-well-plates. After incubation for 12 h, non-migrated cells were removed by scraping. The cells were fixed with 4 % paraformaldehyde for 30 min at 4 °C and stained with crystal violet for 20 min at room temperature. Then, a light inverted microscope (Nikon TE2000-U, Tokyo, Japan) was used to observe the cell migration.

### Cell invasion assay

The cell invasion assay was also conducted using the transwell chambers. The back of chambers was precoated with 5 mg/ml fibronectin (50 μl) and the upper compartment of the polycarbonate filter was coated with Matrigel (5 mg/ml,100 μl). The Matrigel matrix (BD Bioscience, USA) formed a continuous thin layer after drying for 1 h at 37 °C. The OS cells (1 × 10^5^) were put in the upper part of chambers containing 0.1 ml DMEM without serum and 500 μl DMEM containing 10 % FBS were added in 24-well-plates. After incubation for 12 h, non-invaded cells were removed by scraping. The cells were fixed with 4 % paraformaldehyde for 30 min at 4 °C and stained with crystal violet for 20 min at room temperature. Then, a light inverted microscope (Nikon TE2000-U, Tokyo, Japan) was used to observe the cell invasion.

### Real-time quantitative PCR

Total RNA was extracted using RNAiso Plus (Invitrogen, Carlsbad, California, USA) and the RNA samples were reverse-transcribed into cDNA using the Primescript RT reagent Kit (TaKaRa Biotechnology, Dalian, China). The primer sequence for S100A9 was 5′-TGGCTCCTCGGCTTTGACA GAGT-3′(forward) and 5′-TGGGTGCCCCAGCTTCACAGA–3′(reverse), and for GAPDH, 5′-CTTTGGTATCGTGGAAGGACTC-3′(forward) and reverse 5′-GTAGAGGCAGGGATGATGTTCT-3′. Amplification conditions were as follows: incubation at 95 °C for 30s, followed by 40 cycles at 95 °C for 15 s, and finally 60 °C for 45 s. Data was normalized to GAPDH, and mRNA abundance was calculated using the 2^-△△CT^ method [24].

### Western blotting

The cells were harvested and disrupted in lysis buffer (Beyotime Institute of Biotechnology, Beijing, China) containing phosphatase inhibitors and Phenylmethanesulfonyl fluoride (PMSF). An equal amount of each protein sample was separated by 8-12 % SDS-PAGE and transferred to polyvinylidene fluoride (PVDF) membranes. The membranes were blocked with 5 % Bovine Serum Albumin (BSA) and incubated with primary antibodies for overnight at 4 °C, including S100A9(1:800), total ERK1/2 MAPK(1:800), phospho-ERK1/2 MAPK(1:1000), total p38 MAPK (1:600), phospho-p38 MAPK(1:2000), total p65 NF-κB(1:800), phospho-p65 NF-κB(1:1000), total p50 NF-κB(1:500), phospho-p50 NF-κB (1:1000), p21(1:500) and p27(1:500). The membranes were rinsed 10 min for three times with TBST buffer,and incubated with horseradish peroxidase-conjugated secondary antibody (1:5000) for 1 h at 37 °C. Thenthe membranes were rinsed 3 more times with TBST buffer and quantified by the Quantity One 4.6 computer software (Bio-Rad, Hercules, California, USA).

### Enzyme activity assay

The activity assay (Genmed Scientifics Inc, Shanghai, China) of ERK1/2, p38, p65, p50, CDK2 and CDK4 were used to measure the intracellular activity. 50 μg of proteins samples were added into the 96-well-plate and the enzyme activities were determined using an enzyme-labeled instrument at different wavelengths according to the manufacturer’s instruction manual. The values are presented as the percentage (%) of blank control.

### Xenograft tumor model

The male nude mice (4 weeks) were obtained from the experimental animal center of Chongqing Medical University. All mice experiments were approved by experimental animal center of Chongqing Medical University. The OS cells were injected subcutaneously into the nude mice at a density of 5 × 10^6^ cells per 100ul PBS. Tumor volume was measured at 7, 14, 21 and 28 days after injection. Mice were killed at 28 days, the xenograft tumors were dissected and embedded in paraffin for HE staining and IHC.

### Proliferation index in xenograft tumor

IHC staining for the expression of Ki67(1:200) and Proliferating cell nuclear antigen (PCNA)(1:200) in xenograft tumor tissues were conducted. The proliferation index (Ki-67 and PCNA index) was measured (the percentage of positive cells from five randomly fields under a light microscopy at × 400 magnification).

### Statistical analysis

Statistical analyses were performed using SPSS 19.0. Statistical differences among groups were analyzed by ANOVA, *t*-test or chi-square test. The data was presented as mean ± standard deviation (SD). All *p* values were two-sided with statistical significance of *p* < 0.05.

## Results

### Over-expression of S100A9 in human OS tissues

**Fig. 1:**
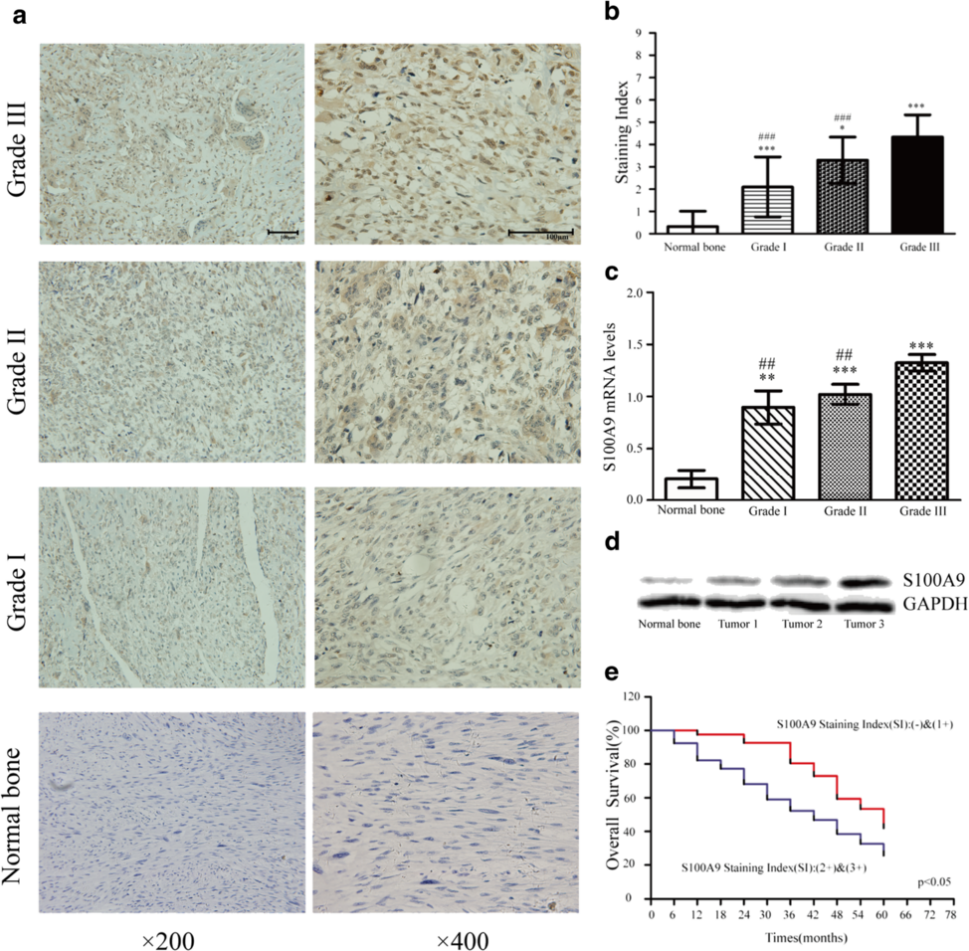
The expression of S100A9 was found in tissue microarrays. **a**. The immunohistochemical analysis of S100A9 expression was performed in 120 human osteosarcoma samples and 40 normal bone samples. Representative cases of OS different grades were shown. **b**. Statistical quantification of SI in normal bone tissues and different grades of OS issues (*p < 0.05, ***p < 0.001, versus normal bone tissues; ###*p* < 0.001, versus OS Grade III). **c**. The mRNA levels of S100A9 were tested by real-time Quantitative PCR in osteosarcoma tissues and normal bone tissues (*n* = 3, **p < 0.01, ***p < 0.001, versus normal bone group; ##*p* < 0.001, versus OS Grade III). **d**. The protein levels of S100A9 were tested in three fresh osteosarcoma tissues and normal bone tissue. Tumor 1 and tumor 2 belonged to grade I, tumor 3 belonged to grade III. **e**. Relationship between overall survival and level of S100A9 expression (*n* = 120, *p* < 0.05)

In this study, we surveyed the expression of S100A9 in human OS tissues and normal human bone tissues, 120 specimens from OS patients and 40 normal human bone tissues were evaluated by tumor tissue microarrays. The histologic subtypes of all OS tissues were originated from osteoblast. Our tissue microarray analyses demonstrated that 95 % of the OS specimens(114 of 120) was positively stained for S100A9 (Table 1). The distribution of S100A9 staining falls into three patterns: nuclear (17.5 %), cytoplasma (20.0 %), and both (57.5 %), but these distribution patterns failed to show a statistical significance on the survival (*p* > 0.05). There were no statistical significances in gender, age, sites according to the staining results (Table 1). Representative specimens with different OS GTM grades staining for S100A9 were shown in Fig. 1a. The data confirmed S100A9 was over-expression in OS and the high-grade tissues presented a higher expression level of S100A9 than low-grade tissues according to the GTM staging system, but there was no statistical significance between Grade I and Grade II (Fig. 1b). The mRNA levels of S100A9 in all tissues were tested by real-time quantitative PCR (Fig. 1c), and the results agreed with the immunohistochemistry. Due to the low incidence of osteosarcoma, we only collected three fresh osteosarcoma tissues to test by western blot (Fig. 1d). We also assessed the survival ratios with respect of S100A9 staining index (SI) in all the human OS patients. 76 of 120 OS patients died at the time of the latest follow-up. We lost contact with 18 of the 120 patients during the follow-ups. Figure 1e demonstrated the survival curves for the human OS patients with S100A9 expression. The risk ratios for those patients with staining scores of moderate group and strong group were greater than those with staining scores of no staining group and weak group (*p* < 0.05).

**Table 1:** Correlation expression of S100A9 in osteosarcoma tissues and normal human bone tissues

Groups	S100A9				*P*-value
	3 + (*n*)	2 + (*n*)	1 + (*n*)	-(*n*)	
Tissue					
Normal tissue (*n* = 40)	0	0	8	32	0.000
Osteosarcoma tissue (*n* = 120)	6	73	35	6	
Gender					
Male (*n* = 71)	4	46	17	4	0.210
Female (*n* = 49)	2	27	18	2	
Age					
> 25 (*n* = 47)	1	28	16	2	0.378
< 25 (*n* = 73)	5	45	19	4	
Site					
Femur (*n* = 73)	4	46	20	3	0.086
Tibiofibula (*n* = 23)	2	15	5	1	
Other sites (*n* = 24)	0	12	10	2	
Clinical stage					
IA (*n* = 25)	0	6	15	4	0.000
IB (*n* = 16)	1	8	5	2	
IIA (*n* = 38)	2	30	6	0	
IIB (*n* = 32)	1	24	7	0	
III (*n* = 9)	2	5	2	0

### Knockdown of S100A9 contributes to reducing OS proliferation, migration and invasion in vitro

**Fig. 2:**
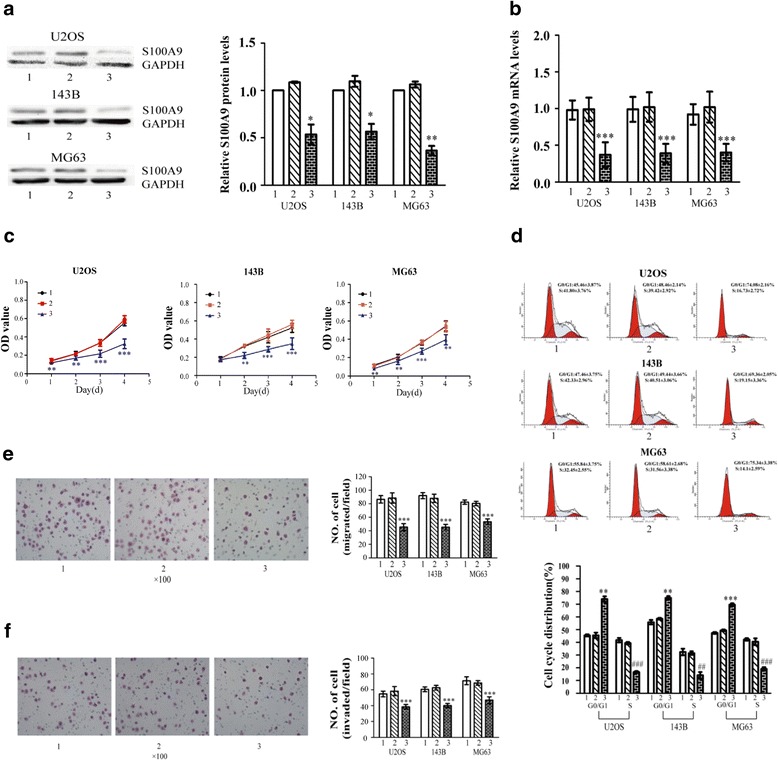
Knockdown of S100A9 contributes to reducing OS proliferation, migration and invasion in vitro. **a**. Western blot was conducted to determine S100A9 protein in the three human OS cell lines (group1-blank control, group2-transfected with empty vectors, group3-transfected with siRNA-S100A9 vectors; *n* = 5, ^*^ *p* < 0.05; ^**^ *p* < 0.01, versus empty vectors group). **b**. Real-time Quantitative PCR was conducted to determine the mRNA levels of S100A9 in the three OS cell lines (*n* = 9, ^***^ *p* < 0.001, versus empty vectors group). **c**. CCK-8 was used to assess the three OS cell lines in 1, 2, 3 and 4 days (*n* = 15,^**^ *p* < 0.01;^***^ *p* < 0.001, versus empty vectors group at the same time points). **d**. Cell cycle distribution was tested in the group1, 2 and 3 using flow cytometry; the histogram of cell cycle distribution was shown (*n* = 5, ^**^ *P* < 0.01,^***^ *P* < 0.001, versus empty vectors group at G0/G1 phase; ^##^ *p* < 0.01, ^###^ *p* < 0.001, versus empty vectors group at S phase). **e**. Cell migration assay were evaluated by transwell chambers in group1, 2 and 3 (*n* = 5, ^***^ *p* < 0.001, versus empty vectors group). **f**. Cell invasion assay were evaluated by transwell chambers in group 1, 2 and 3 (*n* = 5,^***^ *p* < 0.001,versus empty vectors group)

Three OS cell lines (U2OS,MG63,143B) were transfected with S100A9-siRNA. Compared with cells transfected with empty vectors groups and blank control groups, the expression levels of S100A9 protein and mRNA were apparently reduced in the siRNA-S100A9 vectors groups according to the results of western blot (Fig. 2a) and real time PCR (Fig. 2b). CCk-8 assays demonstrated that down-regulation of S100A9 reduced the proliferation of the three OS cell lines in 1, 2, 3 and 4 days (Fig. 2c). Flow cytometric analysis was used for searching the reason why down-regulation of S100A9 could inhibit OS proliferation. The percentage of G0/G1 and S phase cells in each group was shown in Fig. 2d. It was revealed that knockdown of S100A9 could contribute to accumulation of OS cells in G0/G1 phase in comparison with empty vectors groups and blank control groups (Fig. 2d). Next, we assessed the effects of S100A9 knockdown on migration and invasion capacity. The number of cells migrated across the polycarbonate membrane was also reduced after silence of S100A9 (Fig. 2e), while the invasion of tumor cells was significantly inhibited (Fig. 2f). The migration assay and invasion assay proved S100A9 posed a great influence on the metastasis in human OS cell lines.

### Knockdown of S100A9 gene inhibits OS growth in vivo

**Fig. 3:**
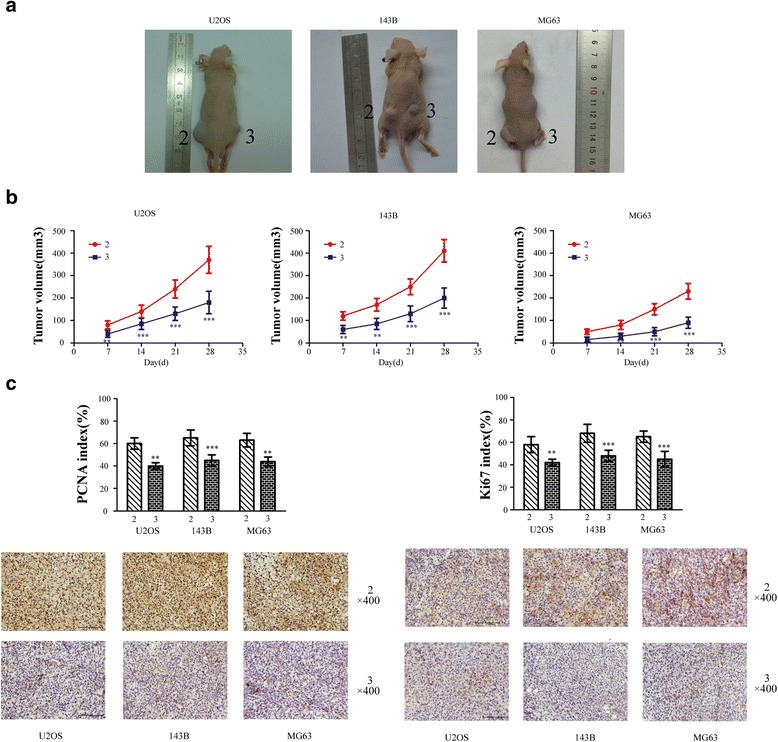
Knockdown of S100A9 gene inhibits OS growth in vivo. **a**. General observation of tumor xenografts in OS cells between empty vectors groups (group2, left) and siRNA-S100A9 vectors groups (group3, right). **b**. The volumes of tumor xenografts in OS cells were measured on a weekly basis and tumor growth curves were plotted (*n* = 5, ^**^ *p* < 0.01; ^***^ *p* < 0.001, versus empty vectors group). **c**. The mice were killed at 28 days and the tissues xenograft tumors were collected to determine the PCNA and Ki67 index (^**^ *p* < 0.01;^***^ *p* < 0.001, versus empty vectors group)

Furthermore, we established the effects of S100A9 on OS growth in vivo using a xenograft model (Fig. 3a). OS cells treated with siRNA- S100A9 vectors groups or empty vectors groups were implanted in subcutaneous tissues of nude mice, and corresponding neoplasm volumes were measured every 7 days. Figure 3b showed that down-regulation of S100A9 in OS cells significantly decreased tumor sizes, compared with empty vectors groups. The proliferating cell nuclear antigen (PCNA) and ki67 proliferation index for these solid tumor masses were calculated after 28 days implantation, the proliferation index of the tumors obtained from siRNA-S100A9 vectors groups was lower than that from the empty vectors groups (Fig. 3c).

### Reduced S100A9 down-regulates MAPK signaling and NF-κB signaling in OS cells

**Fig. 4:**
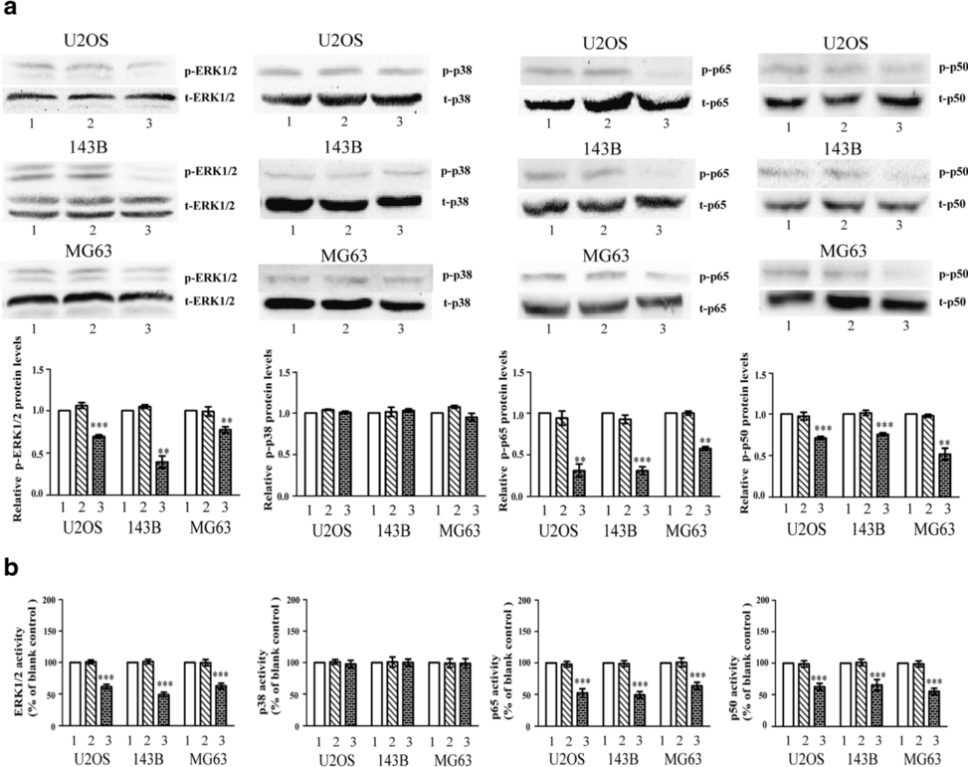
Reduced S100A9 down-regulates MAPK signaling and NF-κB signaling in OS cells. **a**. Three OS cell lines were pretreated with empty vectors and siRNA-S100A9 vectors, and then were tested by western blot in terms of their phosphorylation of ERK1/2 MAPK, p38 MAPK, p50 NF-κB and p65 NF-κB. Total ERK1/2 MAPK, total p38 MAPK, total p50 NF-κB and total p65 NF-κB were used as loading controls (*n* = 5, ^**^ *p* < 0.01; ^***^ *p* < 0.001, versus empty vectors group). **b**. The activity of ERK1/2 MAPK, p38 MAPK, p50 NF-κB and p65 NF-κB in the OS cells of blank groups, empty vectors groups and siRNA-S100A9 vectors groups were quantified by spectrophotometric assay (*n* = 9,^***^ *p* < 0.001, versus empty vectors group)

To probe the molecular events after silence of S100A9 in OS cells, we tested the phosphorylation of MAPK and NF-κB signaling pathways by western blot analysis and enzyme activity assay. Western blot analysis revealed that the protein levels of phospho-ERK1/2 MAPK, phospho-p50 NF-κB and phospho-p65 NF-κB in siRNA-S100A9 vectors groups were lower than those in empty vectors groups and blank control groups in OS cell lines (Fig. 4a). Enzyme activity assay also confirmed the above conclusions (Fig. 4b). However, the protein level and enzyme activity of phospho-p38 MAPK in siRNA-S100A9 vectors groups presented no substantial changes compared with the other two groups (Fig. 4a and b)

### Reduced S100A9 up-regulates the cell cycle-related proteins p21 and p27 causing the inactivation of CDK2 and CDK4

**Fig. 5:**
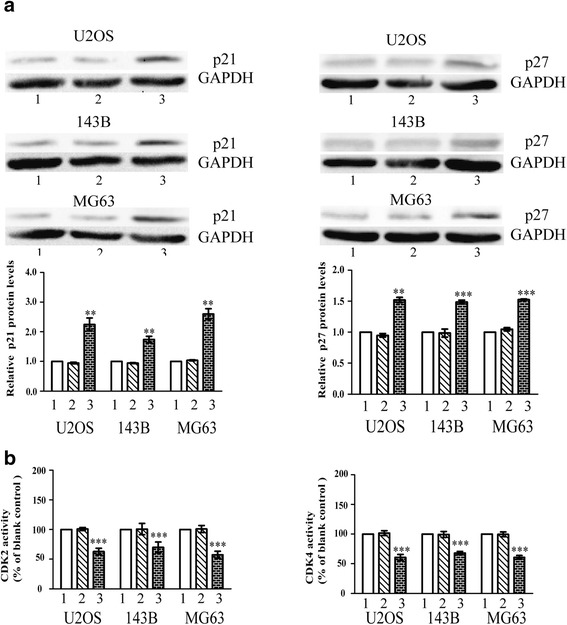
Reduced S100A9 up-regulates the cell cycle-related proteins p21 and p27 causing the inactivation of CDK2 and CDK4. **a**. Three OS cell lines were treated with empty vectors and siRNA-S100A9 vectors, and then were tested by western blot in terms of p21 and p27. GAPDH was used as loading control (*n* = 5, ^**^ *p* < 0.01; ^***^ *p* < 0.001, versus empty vectors group). **b**. The activity of CDK2 and CDK4 in OS cells of blank control groups, empty vectors groups and siRNA-S100A9 vectors groups were quantified by spectrophotometric assay (*n* = 9,^***^ *p* < 0.001, versus empty vectors group)

The inhibition of cell-cycle progression at the G1 checkpoint by cell-cycle regulators p21 and p27 [25] became a considerably attractive mechanism for targeting cancers [26, 27]. Thus, we wondered whether S100A9 modulated the expression of p21 and p27 as well. Western blot quantification revealed an increasing expression of p21 and p27 in siRNA-S100A9 vectors groups (Fig. 5a). In parallel, the enzyme activity assay of cyclin dependent kinase 2(CDK2) and cyclin dependent kinase 4(CDK4) were suppressed in the three OS cells transfected with siRNA-S100A9 (Fig. 5b).

## Discussions

The recent accumulative studies have demonstrated that the S100 family members, notably S100A9, played a critical role in tumor development and progression due to their involvement in survival, growth and metastasis of tumor cells [28]. Nevertheless, little is known concerning the role of S100A9 in human osteosarcoma. In this study, it was the first report that S100A9 was considerably over-expressed in OS patient samples and its expression level in the OS cells was statistically correlated with neoplastic growth. PCNA and ki67 proliferation index would also support these conclusions. Reports showed that the two proteins (PCNA and ki67) have emerged in the S phase and participated in cell proliferation [29]. With the above information, we concluded that the expression level of S100A9 in OS cells could be an independent factor for the patients’ prognosis. All these studies proved that S100A9 was involving in the OS progression.

S100A9, one member of the family of low-molecular-weight intracellular EF-hand motif calcium-binding proteins, is abundantly expressed in cells of the myeloid lineage, including cytoplasmic proteins in neutrophils, macrophages at early-differentiation states and a smaller extent in monocytes [30]. S100A9 which shares a lot of similar characteristics with other S100 proteins locates on a cluster of human chromosome 1q21, where several chromosomal abnormalities have been found to be linked with neoplasia [31–33]. In our study, S100A9 independently promoted the tumor growth, migration and invasion in vitro. But in fact it may be not the only pathway in vivo, the cancer microenvironment might be a critical reason. The composition of tumor microenvironment is very complicated. Infiltrating inflammatory cells have been identified as one important componet. Inflammatory mediators induce the accumulation of myeloid cells, including myeloid-derived suppressor cells (MDSC). They have the potent immunesuppressive effects that could promote tumor growth by inhibiting T-cell-driven anti-tumor immune responses and support immune evasion through the release of reactive oxygen species (e.g. nitric oxide), cytokines, and arginase [34]. Over-expression of S100A9 could increase MDSC recruitment [35]. S100A9 knockout mice are better able to reject EL4 lymphomas compared to wild type mice for inhibiting the recruitment of MDSC [36]. Another example, CD11b(+)Gr1(+) cells are classical markers of murine MDSC which have been identified in tumor-bearing mice [37, 38]. Mice lacking S100A9 have apparently reduced infiltration of CD11b(+)Gr1(+) cells within tumors and pre-metastatic organs showed lower tumor incidence, growth and migration [23]. It has been reported that S100A9 not only induce the accumulation of MDSC, but it is also secreted by MDSC and tumor cells, and bind to cell surface receptors (such as RAGE) leading to MDSC migration [39]. So the autocrine feedback loop is created between S100A9 and MDSC that have significant influence on the inflammatory tumor environment.

In most cases, S100A9 is usually as a ligand. The main receptors for S100A9 are the receptor for advanced glycation mend-products (RAGE) and toll-like receptor 4 (TLR4) [40]. RAGE is a type I transmembrane protein, and a signaling receptor of the immunoglobulin superfamily. Mounting studies have implicated that RAGE involved in many pathologies (diabetes, inflammation, neuronal degeneration and cancers) are regard as a receptor and effector of intracellular responses mediated by DAMPs [41, 42]. Toll-like receptor 4(TLR4) is one of the transmembrane receptors that enable cells of the innate immune system to mount inflammatory responses against pathogen [43]. S100A9 and serum amyloid A3(an important down-stream molecule of S100A9) were bound to TLR4 [44]. RAGE and TLR4 are both implicated in S100A9-mediated tumor-associated pathological effects. It has been reported that S100A9 could recruit additional MDSC into the tumor microenvironment by binding to RAGE [45] and S100A9 interaction with TLR4 is critical for tumor growth in lymphoma [46]. Besides RAGE and TLR4, extracellular matrix metalloproteinase inducer (EMMPRIN), known as CD147, has been found as a new receptor which specifically bound to S100A9 [47]. The carcinogenicity of EMMPRIN might be related to matrix metalloproteinases (MMPs) [48].

In some tumors, S100A9 mediates proliferative and invasive signals and enhances the MAPK or NF-κB signaling pathway [49]. The aberrant activations of MAPK and NF-κB signaling pathways both have critical effects on tumor growth and migration [50–53], which was found in OS cells according to previous studies [54]. Although some components about the downstream of MAPK or NF-κB signaling pathways have been discussed, there were many disputes which genes could control the activity of two signaling pathways. According to our reports, we observed that the suppression of S100A9 caused decreases in the phosphorylation activity of ERK1/2, NF-κB-p50, NF-κB-p65 as well as no detectable phosphorylation of MAPK-p38. This indicated that S100A9 might mediate the tumor progression in OS cells by promoting cellular migration and invasion, which was linked to MAPK and NF-κB signaling pathways. Interestingly, our conclusion was consistent with the findings that S100A9 activated phosphorylation of ERK1/2 but not phosphorylation of MAPK-p38 signaling pathway [23]. Instead, it has been found that S100A9 increased phosphorylation of MAPK-p38 , but inhibited phosphorylation of ERK1/2 in gastric cancers [55]. Therefore, the actions of S100A9 through MAPK signals could be depend on the different types of cancer cells. The MAPK signaling pathways might be act as a various role in different cancerous tumors.

## Conclusions

To sum up, we have evidenced the involvement of S100A9 in OS pathology, showing that knockdown of S100A9 can reduce OS development through inactivation of MAPK and NF-κB signaling pathways. Therefore, we prone to put S100A9 as a significant parameter for predicting human osteosarcoma patients’ prognosis and S100A9 might be used as a potential target for cytokine therapy.

## Availability of data and materials

The datasets supporting the conclusions of this article are available in the repository.

The datasets supporting the conclusions of this article are included within the article and its Additional files 1, 2, 3 and 4.

## Additional files

Additional file 1: FigureS1 The knockdown of S100A9 had no effect on the cell apoptosis, RAGE or TLR4. A. The apoptosis was tested after knockdown of S100A9 (n=3). A histogram about the sum of the upper right quadrant(Q2) and the lower right quadrant(Q3) was shown in Supplement fig.1A. Q2 represents late apoptotic cells, Q3 represents early apoptotic cells. B. The protein levels of RAGE and TLR4 were tested by western blot in three OS cell lines (group1-blank control, group2-transfected with empty vectors, group3-transfected with siRNA-S100A9 vectors; n=3). (TIFF 5193 kb) Additional file 2: FigureS2 The up-regulation of S100A9 enhanced osteosarcoma proliferation, migration and invasion.A.The protein levels of S100A9 were tested by western blot in the three OS cell lines (group1-blank control, group2-transfected with empty vectors, group3-transfected with S100A9 vectors; n=3, ***P < 0.001,versus empty vectors group). B. The mRNA levels of S100A9 were tested by real-time Quantitative PCR in the three OS cell lines (n=9, ***P < 0.001, versus empty vectors group). C. CCK-8 was used to assess the three OS cell lines in 1, 2, 3 and 4 days (n=15, *p < 0.05, **P < 0.01; ***P <  0.001, versus empty vectors group at the same time points). D. Cell cycle distribution was tested in the group1, 2 and 3 using flow cytometry; the histogram of cell cycle distribution was shown(n=3, **P < 0.01, versus empty vectors group at G0/G1 phase; ##P < 0.01, versus empty vectors group at S phase). E. The histogram of cell migration assay were evaluated by transwell chambers in group1, 2 and 3(n=5,***P < 0.001, versus empty vectors group). F. The histogram ofcell invasion assay were evaluated by transwell chambers in group1, 2 and 3 (n=5, ***P < 0.001, versus empty vectors group).(TIFF 10616 kb) Additional file 3: FigureS3 A.The mRNA levels of S100A9 were tested by real-time Quantitative PCR in osteosarcoma tissues andnormal bone tissues(n=3, **p < 0.01, ***P < 0.001, versus normal bone group; ##p < 0.001, versus OS Grade III). B.The protein levels of S100A9 were tested in three fresh osteosarcoma tissues and normal bone tissue. Tumor 1 and tumor 2 belonged to grade I, tumor 3 belonged to grade III.(TIFF 1805 kb) Additional file 4: FigureS4 A.The photos of immunofluorescence for observing in the OS cell lines were shown. B. CCK-8 was used toassess the three OS cell lines in 1, 2, 3 and 4 days (n=15, group1-blank control, group2-transfected with empty vectors, group3-transfected with siRNA-TLR4 vectors). C. CCK-8 was used to assess the three OS cell lines in 1, 2, 3 and 4 days (group1-blank control, group2-transfected with empty vectors, group3- 10.1186/s12885-016-2294-1transfected with siRNA-EMMPRIN vectors; n=15, **P < 0.01; ***P < 0.001)(TIFF 4998 kb)

## Abbreviations

CCK8: cell counting kit-8
CDK2: cyclin dependent kinase 2
CDK4: cyclin dependent kinase 4
DMEM: Dulbecco’s modified Eagle’s medium
EMMPRIN: extracellular matrix metalloproteinase inducer
FBS: fetal bovine serum
GAPDH: glyceraldehydes-3-phosphate dehydrogenase
MAPK: mitogen-activated protein kinase
MDSC: myeloid-derived suppressor cells
NF-κB: nuclear factor-κB
OS: osteosarcoma
PCNA: proliferating cell nuclear antigen
PCR: polymerase chain reaction
PMSF: phenylmethanesulfonyl fluoride
PVDF: polyvinylidene difluoride
RAGE: receptor for advanced glycation mend-products (RAGE)
TLR4: toll-like receptor 4
