# Supplementary figures and images for: Down-regulation of S100A9 inhibits osteosarcoma cell growth through inactivating MAPK and NF-κB signaling pathways
Source: BMC Cancer. 2016 Mar 28;16:253. doi: 10.1186/s12885-016-2294-1 (PMC4810516; doi:10.1186/s12885-016-2294-1)

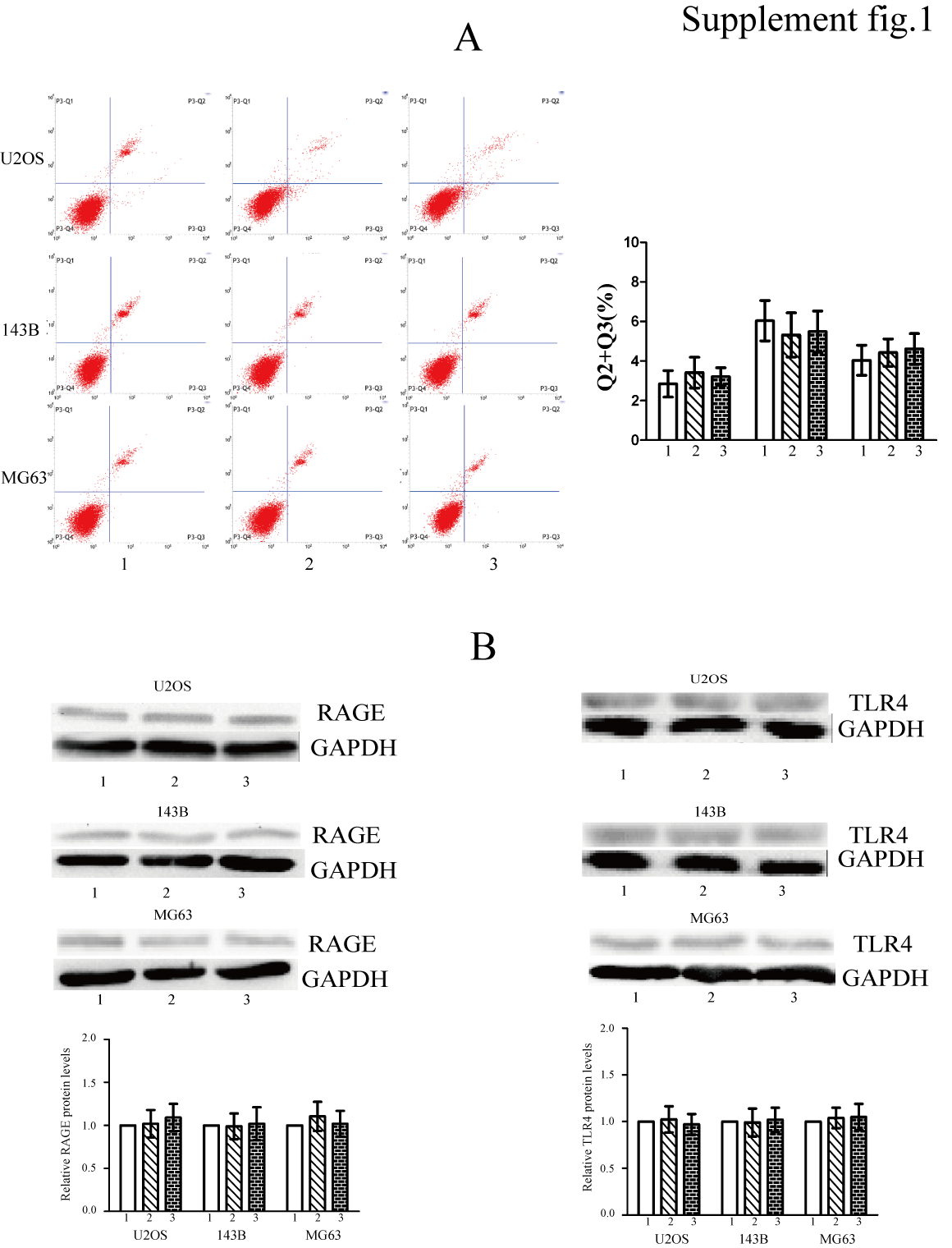

Supplement: Additional file 1: FigureS1 — The knockdown of S100A9 had no effect on the cell apoptosis, RAGE or TLR4. A. The apoptosis was tested after knockdown of S100A9 (n=3). A histogram about the sum of the upper right quadrant(Q2) and the lower right quadrant(Q3) was shown in Supplement fig.1A. Q2 represents late apoptotic cells, Q3 represents early apoptotic cells. B. The protein levels of RAGE and TLR4 were tested by western blot in three OS cell lines (group1-blank control, group2-transfected with empty vectors, group3-transfected with siRNA-S100A9 vectors; n=3). (TIFF 5193 kb) [file 12885_2016_2294_MOESM1_ESM.tiff]

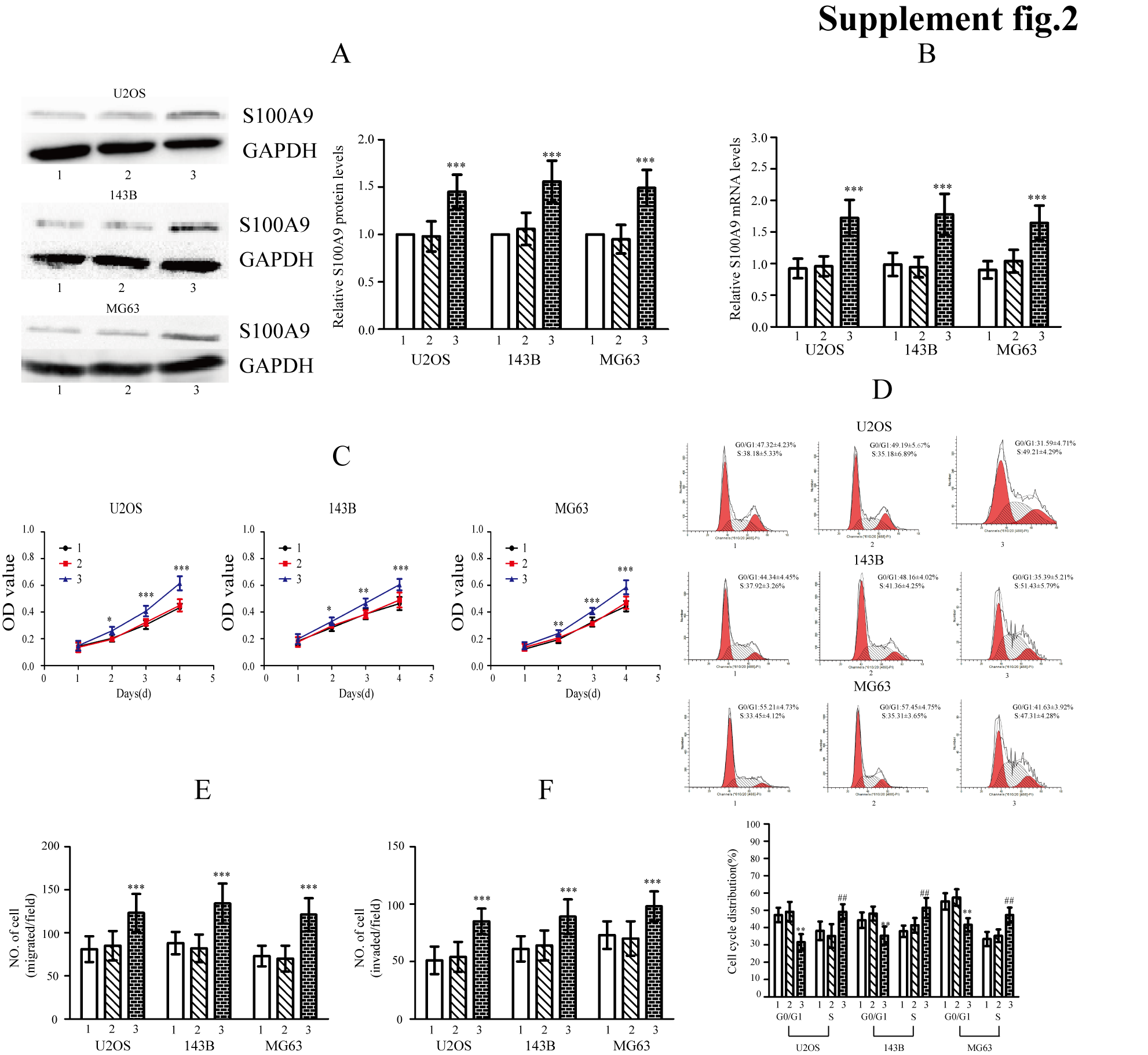

Supplement: Additional file 2: FigureS2 — The up-regulation of S100A9 enhanced osteosarcoma proliferation, migration and invasion.A.The protein levels of S100A9 were tested by western blot in the three OS cell lines (group1-blank control, group2-transfected with empty vectors, group3-transfected with S100A9 vectors; n=3, ***P < 0.001,versus empty vectors group). B. The mRNA levels of S100A9 were tested by real-time Quantitative PCR in the three OS cell lines (n=9, ***P < 0.001, versus empty vectors group). C. CCK-8 was used to assess the three OS cell lines in 1, 2, 3 and 4 days (n=15, *p < 0.05, **P < 0.01; ***P < 0.001, versus empty vectors group at the same time points). D. Cell cycle distribution was tested in the group1, 2 and 3 using flow cytometry; the histogram of cell cycle distribution was shown(n=3, **P < 0.01, versus empty vectors group at G0/G1 phase; ##P < 0.01, versus empty vectors group at S phase). E. The histogram of cell migration assay were evaluated by transwell chambers in group1, 2 and 3(n=5,***P < 0.001, versus empty vectors group). F. The histogram ofcell invasion assay were evaluated by transwell chambers in group1, 2 and 3 (n=5, ***P < 0.001, versus empty vectors group).(TIFF 10616 kb) [file 12885_2016_2294_MOESM2_ESM.tiff]

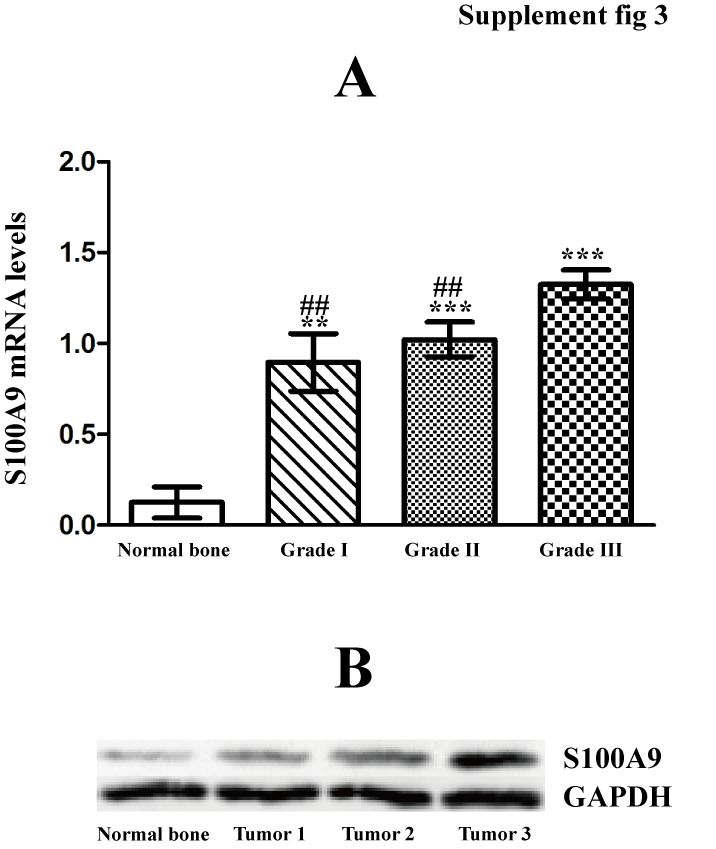

Supplement: Additional file 3: FigureS3 — A.The mRNA levels of S100A9 were tested by real-time Quantitative PCR in osteosarcoma tissues andnormal bone tissues(n=3, **p < 0.01, ***P < 0.001, versus normal bone group; ##p < 0.001, versus OS Grade III). B.The protein levels of S100A9 were tested in three fresh osteosarcoma tissues and normal bone tissue. Tumor 1 and tumor 2 belonged to grade I, tumor 3 belonged to grade III.(TIFF 1805 kb) [file 12885_2016_2294_MOESM3_ESM.tiff]

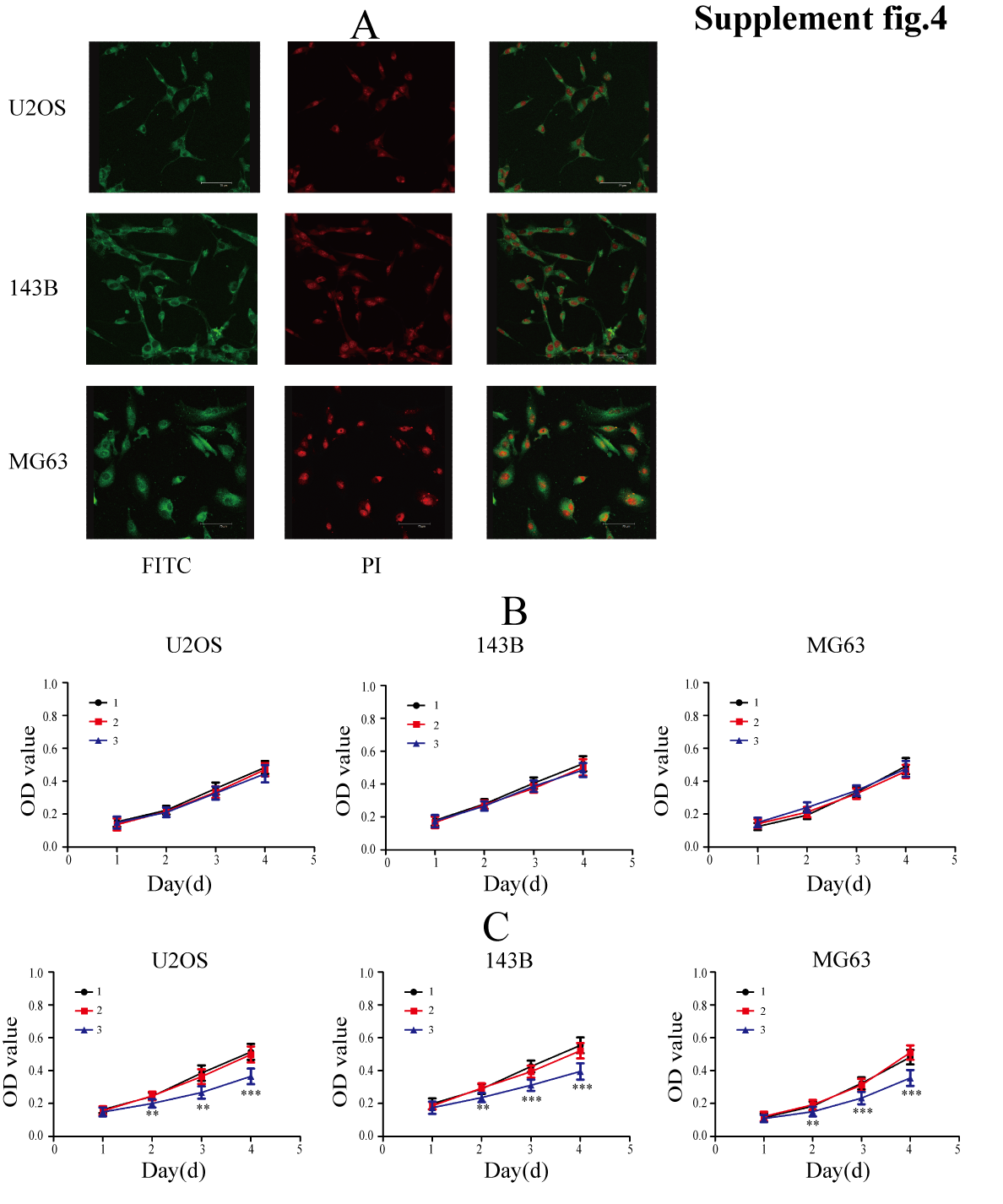

Supplement: Additional file 4: FigureS4 — A.The photos of immunofluorescence for observing in the OS cell lines were shown. B. CCK-8 was used toassess the three OS cell lines in 1, 2, 3 and 4 days (n=15, group1-blank control, group2-transfected with empty vectors, group3-transfected with siRNA-TLR4 vectors). C. CCK-8 was used to assess the three OS cell lines in 1, 2, 3 and 4 days (group1-blank control, group2-transfected with empty vectors, group3- 10.1186/s12885-016-2294-1transfected with siRNA-EMMPRIN vectors; n=15, **P < 0.01; ***P < 0.001)(TIFF 4998 kb) [file 12885_2016_2294_MOESM4_ESM.tiff]
